# Attention without Constraint: Alpha Lateralization in Uncued Willed Attention

**DOI:** 10.1523/ENEURO.0258-22.2023

**Published:** 2023-06-09

**Authors:** John G. Nadra, Jesse J. Bengson, Alexander B. Morales, George R. Mangun

**Affiliations:** 1 Center for Mind and Brain, University of California, Davis, Davis, California 95618; 2 Department of Psychology, University of California, Davis, Davis, California 95616; 3 Department of Neurology, University of California, Davis, Davis, California 95817

**Keywords:** attention, attentional control, EEG, machine learning, volition, will

## Abstract

Studies of voluntary visual spatial attention have used attention-directing cues, such as arrows, to induce or instruct observers to focus selective attention on relevant locations in visual space to detect or discriminate subsequent target stimuli. In everyday vision, however, voluntary attention is influenced by a host of factors, most of which are quite different from the laboratory paradigms that use attention-directing cues. These factors include priming, experience, reward, meaning, motivations, and high-level behavioral goals. Attention that is endogenously directed in the absence of external attention-directing cues has been referred to as “self-initiated attention” or, as in our prior work, as “willed attention” where volunteers decide where to attend in response to a prompt to do so. Here, we used a novel paradigm that eliminated external influences (i.e., attention-directing cues and prompts) about where and/or when spatial attention should be directed. Using machine learning decoding methods, we showed that the well known lateralization of EEG alpha power during spatial attention was also present during purely self-generated attention. By eliminating explicit cues or prompts that affect the allocation of voluntary attention, this work advances our understanding of the neural correlates of attentional control and provides steps toward the development of EEG-based brain–computer interfaces that tap into human intentions.

## Significance Statement

Understanding how behavioral goals influence how we allocate our voluntary attention is a central aim in cognitive neuroscience. A dominant paradigm for studying voluntary attention uses external cues (e.g., arrows) to focus spatial attention. However, real-world attention can be oriented purely by self-initiated volitional processes, known as “willed attention.” We used a novel paradigm that allowed participants the freedom to choose where and when to attend within an ongoing stimulus stream, eliminating potential external biases imposed by cues. We used support vector machine decoding of EEG alpha signals to investigate the temporal dynamics of willed attention shifts as volunteers made self-initiated shifts of spatial attention. Such an approach permits the investigation of the neural correlates of purely voluntary attention.

## Introduction

William James famously wrote, “Everyone knows what attention is. It is the taking possession by the mind, in clear, and vivid form, of one of what seems several simultaneously possible objects or trains of thought” (James, 1890). Attention is the cognitive ability that allows humans to ignore irrelevant stimuli and focus on the most relevant sensory inputs. It can be controlled by either top-down (goal-directed or voluntary) or bottom-up (sensory or reflexive) influences (Posner, 1980; Jonides, 1983; Corbetta and Shulman, 2002; Bowling et al., 2020).

The ability to exert voluntary control over the focus of our attention is arguably a key component of the integrated sense of being that humans experience (Posner, 1994). For decades, voluntary attention has been effectively studied in humans in attention-cuing paradigms using behavioral, electroencephalographic (EEG), and neuroimaging methods (Posner et al., 1980; Harter et al., 1989a,b; Mangun and Hillyard, 1991; Luck et al., 1996; Corbetta et al., 2000; Hopfinger et al., 2000; Gayet and Peelen, 2022). In such paradigms, the experimenter determines how the observer will allocate their attention by manipulating their expectancy about when, where and/or what an upcoming task-relevant target will be (Posner et al., 1980; Müller and Rabbitt, 1989; Kingstone, 1992), or explicitly instructing the observer how to focus attention on each trial (Hopfinger et al., 2000; Hopf and Mangun, 2000; Lanssens et al., 2022).

In everyday vision, however, *voluntary* attention is influenced by many factors, most of which are quite different from the highly controlled cuing paradigms used in the laboratory. When attention is voluntarily directed in the absence of explicit external cues, this has been referred to as internally driven (Taylor et al., 2008) or self-initiated (Hopfinger et al., 2010) attention, or as in our prior work, as “willed attention” (Bengson et al., 2014; 2015; Liu et al., 2017; Rajan et al., 2018; Bengson et al., 2020). The idea in willed attention is that volition drives attention in a manner analogous to how volition initiates motor actions in studies of movement intention (Libet et al., 1983b; Haynes et al., 2007; Haggard, 2008; Soon et al., 2008); but it is arguably, theoretically dissociable (Searle, 1980). Willed attention is expected to be of particular utility when behavioral goals are in conflict with bottom-up salience and other attention-biasing influences (Bacon and Egeth, 1994; Lavie, 2005; Mevorach et al., 2010; Theeuwes, 2018).

Prior investigations of willed attention were derived from standard spatial attention-cuing paradigms that were modified to include prompts that simply signaled the subject to make a willful choice about where to attend on that trial (Taylor et al., 2008; Hopfinger et al., 2010; Bengson et al., 2014; Trachel et al., 2015). While not an attention-directing cue, such prompts are nonetheless artificial laboratory stimuli that instruct the subject to do something in that instant; that is, to make a free choice about where to attend. The present experiment used a novel experimental paradigm that included no cues or prompts. Instead, the subjects viewed ongoing streams of stimuli in the two visual fields and were asked to direct their covert spatial attention to the stream in one hemifield at a time and to a location of their own choosing. The goal of the study was to eliminate any cue/prompt, even a temporal one, from the task and to investigate the neural correlates of this attention control using EEG measures and machine learning decoding methods.

The fact that attention-directing cues and prompts are part of artificial laboratory methodology does not, on the face of it, mean they are problematic. But, one cannot rule out that attentional cues may introduce distortions in the allocation of attention, perhaps by introducing additional cognitive processes, and/or by altering our laboratory measures of attention. For example, as noted by Gmeindl et al. (2016), attention-directing cues and prompts may elicit expectancy, and therefore anticipatory attention, for the appearance of these items. As well, cues and prompts may engage other perceptual or motor processes (e.g., response preparation). In addition, some forms of attention-directing cues have also been shown to induce reflexive attentional orienting. Studies by Ristic et al. (2002) and Ristic and Kingstone (2006, 2012) showed that arrows (a commonly used attention-directing cue in voluntary cue–target paradigms) can trigger a reflexive orienting of attention, because of their overlearned, and therefore prepotent, influences on attention. Yet another concern is that attention-directing cues (and perhaps also prompts) may alter measures of spatial attention (e.g., alpha EEG measures), as argued by Antonov et al. (2020) in their critique of the study by Gundlach et al. (2020). Although our willed attention paradigm does not directly compare instructed (cued) attention to willed attention, the paradigm used here eliminates all of these potentially confounding issues arising from the use of attention-directing cues or prompts, thereby permitting a purer test of the cognitive–neural mechanisms of voluntary attention.

## Materials and Methods

### Participants

EEG data were recorded from 30 undergraduate student volunteers (20 females) at the University of California, Davis. All participants had normal or corrected-to-normal vision, gave informed consent, were screened for neuropsychiatric conditions, and were paid for their participation. One subject was excluded for an inability to follow the task instructions, three subjects were removed for a technical issue with data collection, and four subjects were excluded for excessive EEG artifacts contaminating >25% of their data. Two additional subjects were excluded because they had no trials remaining in at least one data bin after artifact rejection. Thus, the final analysis was conducted on 20 right-handed subjects who met all inclusion criteria.

### Paradigm and stimuli

**Figure 1.**
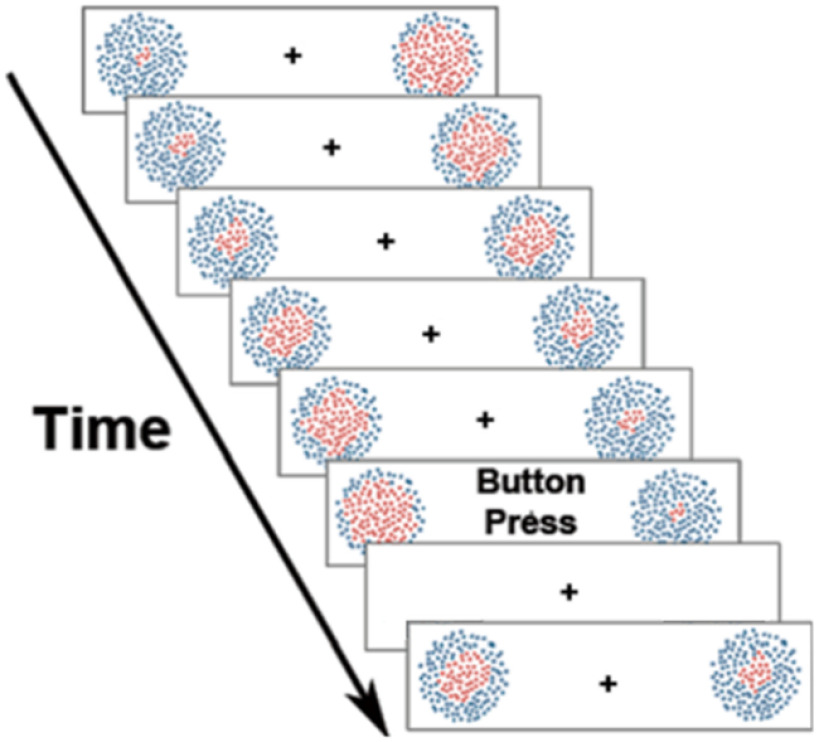
Diagrammatic representation of the dynamic stimulus arrays. Each panel in the figure represents a selected (not immediately sequential) screen refresh. When the sequence ended, there was a constant 500 ms delay between the offset of the trial and the onset of the next trial.

Each trial began with the presentation of a circular patch of 250 red and blue dots in each hemifield (Fig. 1). Each patch of dots had a radius of 5° of visual angle, and each dot was ∼0.23° of visual angle. Each patch was located on the horizontal meridian, ∼4° (to center) lateral to fixation. To enable the possible analysis of focused attention using the steady-state visual evoked potential (SSVEP) method (Müller et al., 1998), in one hemifield flickered on and off continuously at 4 Hz, while those in the other flickered at 6 Hz. From trial to trial, on a random basis, the frequency of flicker in the left and right patches varied (one patch always 4 Hz, and the other 6 Hz); the SSVEP data are not, however, considered in this report. The dots varied randomly in position by 0.08° every one to three screen refreshes (16.67 ms), which induced the perception of continuous random motion. In addition, within each hemifield the proportion of red to blue dots varied in a systematic and continuous fashion from a minimum of 20 red dots in the center with 230 blue dots in the surround to a maximum of 230 red dots in the center and 20 red dots in the surround. With each screen refresh, the number of red dots increased by four as a growing circle, and the number of the blue dots decreased by four as a decreasing annulus; this created the impression of an expanding circle of red dots within the field of blue dots in each circular patch. Once the red dot number reached the maximum of 230 red dots, the pattern changed directions so that red dots started to decline (being replaced by blue). The perception of dot patches is of a continuously expanding and contracting circle of red dots within each circle. Given a 16.67 ms refresh rate, the time from minimum to maximum in the number of red dots was ∼1.0 s, but, on average, each trial lasts ∼4 s, because subjects could begin covert attention at varying intervals after the onset of the array. The expansion/contraction of the red dots in the left and right hemifields occurred asynchronously, so that it was not possible to predict what the pattern in one hemifield was doing given that in the other hemifield. After a button press, the patches disappeared for 500 ms, and the next trial began when the patches reappeared. The fixation point remained on the screen for the duration of each block.

### Procedure

Participants were instructed to maintain ocular fixation on the center cross and to not deviate their eyes during each trial. Once the bilateral stimulus arrays appeared, their task was to voluntarily shift and focus covert attention on one side of the bilateral display at a time of their own choosing. Then, after selectively attending one side, they were to covertly monitor the attended stimulus to detect when the proportion of red dots in the patch reached its maximum, and to push a handheld button in response. They were urged to deploy their attention whenever they wished, and to maintain covert attention until the trial was completed. Importantly, they were told not to use any explicit strategy or develop any pattern for choosing when or to which side to deploy covert attention (e.g., alternating sides on each trial), and to not decide before trial onset which hemifield patch to attend. In other words, once the bilateral array appeared, the subjects were requested to make a spontaneous decision about which side on which to focus covert spatial attention. The participants were told to maintain their attention on the chosen hemifield patch for at least one full expansion cycle of the red dots (∼1 s) while trying to discriminate the maximum size of the expanding red dots in the chosen hemifield. Speeded button presses were required when the maximal red dot expansion was detected. Responses were made with their right hand, pushing the left arrow on a keyboard with their index finger if they had chosen to attend left on that trial, or pushing the right arrow with their middle finger if they had chosen to attend right. Thus, the button responses not only signaled the time of their detection of the target, but also provided feedback as to whether they were attending left or right on that trial. The subjects were told to completely ignore the opposite (unattended) hemifield patch.

### EEG recording and analysis

The EEG was recorded from 64 tin electrodes mounted in an elastic electrode cap (Electro-Cap International) at the following scalp locations: Fp1, Fp2, F7, F3, Fz, F4, F8, FC5, FC1, FC2, FC6, T7, C3, Cz, C4, T8, CP5, CP1, CP2, CP6, P7, P3, Pz, P4, P8, PO9, O1, Oz, O_2_, PO10, AF7, AF3, AF4, AF8, F5, F1, F2, F6, FT9, FT7, FC3, FC4, FT8, FT10, C5, C1, C2, C6, TP7, CP3, CPz, CP4, TP8, P5, P1, P2, P6, PO7, PO3, POz, PO4, and PO8 (Oostenveld and Praamstra, 2001). The EEG was amplified with a Neuroscan Synamps II amplifier (Compumedics). These sites were referenced to FCZ during recording but were rereferenced offline to the algebraic average of TP9 and TP10 (adjacent to the left and right mastoids). The continuous EEG was recorded with a bandpass filter of DC 100 Hz and digitized at 1000 samples/s, and then downsampled offline to 250 samples/s. Before artifact rejection, a bandpass filter for 0.05–50 Hz was applied to the data. Eyeblinks were removed using independent component analysis methods (Vigário, 1997). Residual artifacts were detected automatically, and trials with excessive artifacts were removed using the ERPLAB moving window peak-to-peak artifact rejection (threshold, 100 μV), iterating through the data with a moving window of 100 ms in 50 ms steps. An additional moving window approach was applied to channels FT9 and FT10 to ensure that no trials with eye movements were left in the data. The parameters for this additional moving window approach marked all trials that exceeded 20 μV within a sliding 100 ms window, across 50 ms steps. Each epoch was also visually inspected to manually reject artifacts not picked up by the prior methods, as well as to verify that the artifact rejection pipeline was functioning as intended. The data were then epoched in two separate time periods: −1000 to 4000 ms relative to the onset of the sequence, as well as −4000 to 1400 ms relative to the button press. There are no baseline periods for either of these analyses, considering the constantly present and transient nature of the stimuli. Preprocessing was conducted using both the EEGLAB (Delorme and Makeig, 2004) and ERPLAB (Lopez-Calderon and Luck, 2014) plugins for MATLAB.

In line with our prior work on willed attention (Bengson et al., 2014), we focused our analyses on the alpha band of the EEG. To examine the onset and strength of the willed attention signal, we extracted the trial-by-trial alpha band signal relative to the following two distinct time points: (1) the onset of the sequence and moving forward in time; as well as (2) moving backward in time from the onset of the button press, which logically followed willed shifts of covert attention. We then used the direction of the decision to attend as a grouping variable, labeling trials relative to whether the participant chose to attend to the left or right hemifield on a trial-by-trial basis. For these analyses, the time–frequency analysis was performed on each trial via a short sliding Hanning taper with an adaptive time window of three cycles at each frequency, conducted from 9 to 11 Hz, then the frequencies were averaged together. The alpha frequency band analysis was placed at 9–11 Hz to minimize overlap with the 4 and 6 Hz background flickering of the stimulus arrays. The Fourier analyses were conducted using the fieldtrip toolbox plugin for MATLAB (Oostenveld et al., 2011).

We implemented a support vector machine (SVM) decoding pipeline that was similar to that used by Bae and Luck (2018). The fitcsvm function in MATLAB was used to carry out this analysis. A threefold cross-validated support vector machine was trained and tested separately over each individual time point (in 20 ms increments). The cross-validation that was implemented allowed the same data to act as both the training and the testing sets. The data were averaged within each cross-fold during this process. The data were split into three equal portions, where, in the first iteration, two-thirds are used for training, then one-third is used for testing. On the next iteration, the training and testing sets are randomized to test the classifier across varying subsets of the data. This process was repeated across 10 iterations, then the accuracies obtained from the testing set were averaged across iterations for each time point and averaged over trials. Nineteen relevant electrode channels were included (all parietal and occipital electrodes), and the data were Fourier transformed (extracting alpha band signals at 9–11 Hz) before the training and testing of the SVM. The classification was a binary SVM, computing the classification accuracy of trials where participants were deploying attention to the left versus the right. Given that the trial count (i.e., attending left vs right) is potentially variable given the self-generated decisions about where to attend, each participant’s trial count per bin (left/right) was set to equal trial lengths by randomly shuffling the trial index and dropping trials from the larger bin. We used a nonparametric cluster-based Monte Carlo simulation technique (similar to the commonly used cluster-based mass univariate approach). This method was chosen because of its correction for multiple comparisons and the fact that decoding accuracy may not be normally distributed. The decoding accuracy was extracted at each time point, then tested with a one-sample *t* test (one sample, as below chance decoding is not relevant to our findings). We searched for significant clusters where the *t* tests were significant (*p* < 0.05), and then the *t* scores were combined to create a cluster-level *t* score. Then we assessed whether the cluster *t* score was higher than the *t* score expected by chance (generated by the Monte Carlo simulation), which controls the type 1 error rate at a cluster level. Then, each simulated trial was a randomly sampled number (whether 1 or 2) to compute the chance level for each bin (left or right). The Monte Carlo technique had 10 iterations, with three validations, indicating that this process was repeated 60 times (2 bins × three cross-validations × 10 iterations). This process was then repeated once for each time point (201 time points for the sequence onset decoding; 176 for the button press decoding) to find an accurate decoding accuracy at each datapoint. The data were then smoothed over five time points for graphing purposes. This process was repeated for the data of each of our participants.

## Results

### Trial response latency

**Figure 2.**
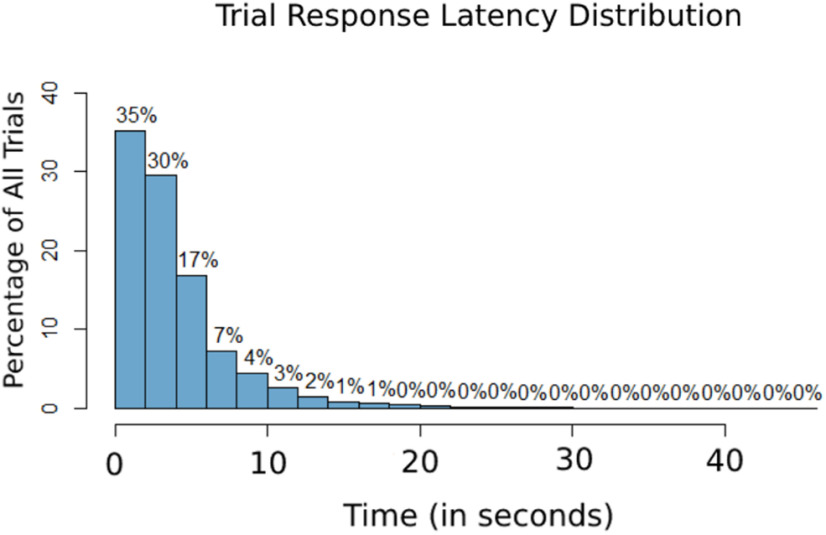
The distribution of the trial response latencies (from trial onset to button press terminating the trial). The *x*-axis is the time in seconds, and the *y*-axis is the percentage of all trials presented.

The mean trial response latency—as measured from trial onset to trial termination (Fig. 2)—was 3945 ms for trials where attention was deployed to the left (SD* *=* *3786), and 3908 ms when deployed to the right (SD* *=* *3701). The difference between these trial response latencies was not statistically significant in a two-sample *t* test (*p* = 0.6289; 95% CI = −113.012, 186.978). Given the task design (unattended-sided stimuli were to be completely ignored), there are no behavioral measures of selective spatial attention (i.e., attended vs unattended reaction times); however, we have two measures of their lateralized spatial attention. The first is that they responded to the targets with either the left or right button, indicating that they were attending left or right on that trial, respectively. The second is the physiological measures of lateralized attention in the form of the well known alpha lateralization with spatially lateralized attention; we present an alpha-band topographic analysis below (see Fig. 4), which provides evidence that participants were indeed focusing spatial selective attention in this task. In terms of how many times participants chose to attend to each side, participants reported (via their left or right button press) that they chose to covertly attend the left hemifield patch 4924 times in total, while the right hemifield patch was attended 4653 times across all participants. There are a total of 9577 trials included in these analyses across all subjects. To examine whether the previous trial influenced decision outcomes, a logistic regression generalized linear model was fit to the behavioral data, which solidified that the previous trial did not have a significant influence on the direction to which attention was shifted on any given trial (*p* = 0.3375).

### Target reaction time

**Figure 3.**
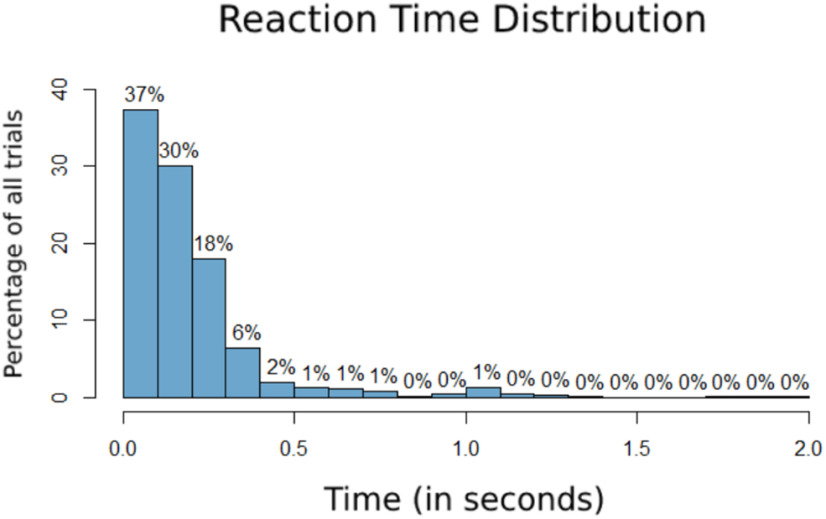
The distribution of reaction times relative to the target (maximal expansion of the attended side). The *x*-axis is the time in seconds, and the *y*-axis is the percentage of all trials presented.

The distribution of reaction times relative to the maximal expansion of the attended side (Fig. 3). The mean reaction time of trials where attention was deployed to the left was 195 ms (SD* *=* *214), whereas when attention was deployed to the right it was 186 ms (SD* *=* *209). We also analyzed whether reaction times were within 500 ms of the maximal expansion to assess how temporally accurate participants were in aligning their responses to the attended target. We found that participants were within 500 ms of the target expansion 93% of the time, thus implying that they were adequately performing the task as described. The few trials (<1%) nearing 2 s reaction times are likely artifacts where participants responded to the stimulus too late to be counted for the current trial. We also conducted a two-sample *t* test between reaction times for the right and left hemifields, but did not find significance (*p* = 0.0883, 95% CI = −0.0206, 0.0014).

### Alpha-band oscillations

**Figure 4.**
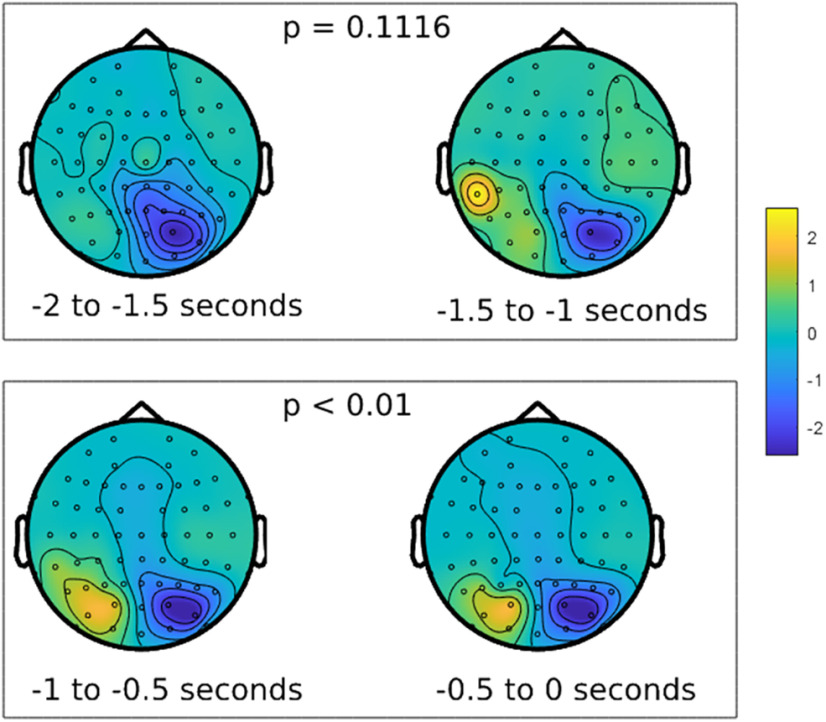
Alpha-band (9–11 Hz) difference plot (attend left – attend right) during two distinct time periods preceding the button press to mark the active focus of attention to the chosen hemifield. The top panel shows the time periods from −2 to −1 s before the button press (in 500 ms sections), while the bottom panel shows the time period directly preceding the report, from 1 s before the button press to the onset of the button press itself (in 500 ms sections). The color scale is based on the absolute value relative to the highest/lowest difference in power across both plots.

We began by validating our task to ensure that participants had allocated selective visual spatial attention in our design. To do this, we relied on the well established EEG alpha correlates of focused visual spatial attention, which show left versus right posterior scalp EEG alpha power asymmetries with spatial attention to lateral visual field locations (Table 1; Worden et al., 2000; Rihs et al., 2007; Romei et al., 2010; Bengson et al., 2014; Liu et al., 2016). We compared the distribution of raw alpha power across the left and right posterior scalp for the choose-left and choose-right trials across all electrodes (Fig. 4). We found significant (two-sided *t* test) left versus right alpha power asymmetries over posterior scalp in the 1000 ms before the button press (*p* < 0.01, 95% CI = −0.442, −0.107); this pattern was not significant in an earlier time window from −2000 to −1000 ms before the button press (*p* = 0.1116, 95% CI = −0.269, 0.028). This alpha power lateralization with spatial selective attention demonstrated that alpha band oscillations serve as a reliable index of the direction of covert spatial attention in our willed attention design. To ensure that the oscillatory signal we derived from the data are real oscillations rather than fractal nonoscillatory components, we conducted irregular resampling autospectral analysis (Wen and Liu, 2016), which showed that real oscillations are prevailing over nonfractal components. With this expected result firmly established, we turned to decoding the time course of the allocation of willed attention.

**Table 1:** *t* Test results for attend left versus attend right in EEG alpha band activity (9–11 Hz)

Time (s)	*p*-value	Confidence interval	df	*t*
−2 to −1	0.1116	−0.269, 0.028	2655.4	−1.5916
−1-0	0.001308	−0.442, −0.107	2644.5	−3.2176

### Decoding results

**Figure 5.**
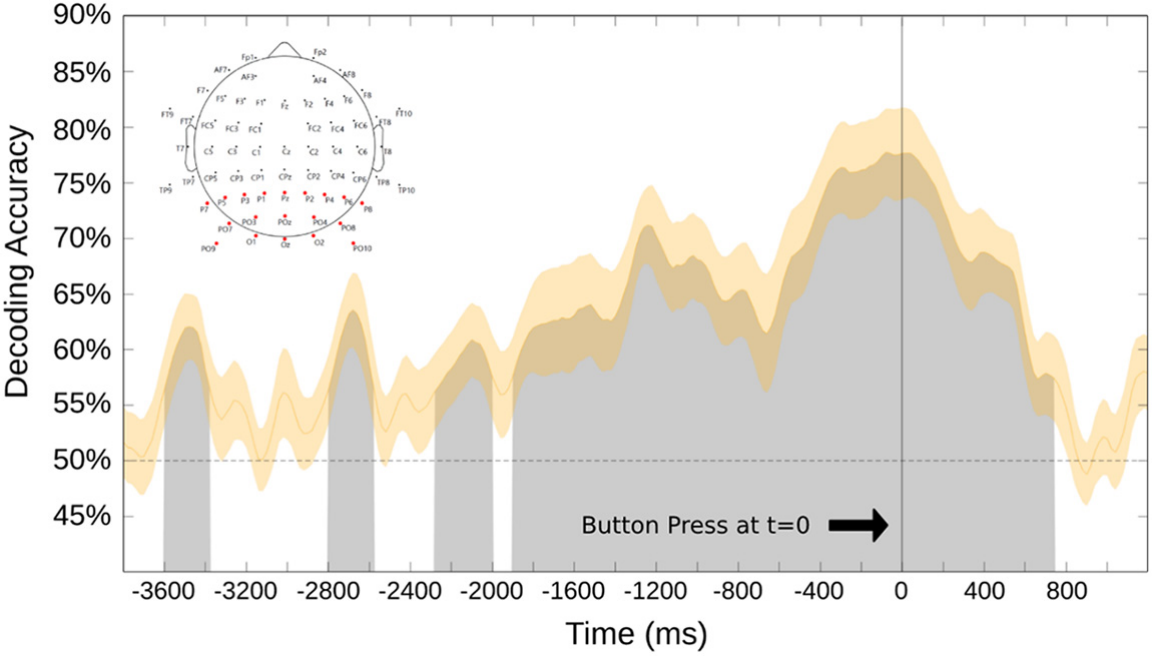
The support vector machine decoding accuracy at each time point, time locked (*t* = 0) to the button press signaling sustained attention to one hemifield. The shaded area surrounding the curve is the SE. This analysis was done over 19 occipital and parieto-occipital electrodes (shown as the red electrodes in the map at top left). Black line denotes time 0, which is the recorded onset of the button press.

Figure 5 shows the decoding accuracy for attend-left versus attend-right during willed attention over 19 occipital and parieto-occipital electrodes, collapsed across the 20 participants in the study. These support vector machine classifier results are for the data time locked to the onset of the button press (*t* = 0 ms). Decoding accuracy starts at chance level (dashed line) and rises above chance over time. The classifier results show statistically significant, robust and consistent decoding of EEG alpha oscillations over time from approximately −1900 ms before button press; the classifier could accurately decode attend-left from attend-right choices until ∼750 ms after the button press. Before approximately −1900 ms, decoding accuracy is lower and more sporadic, which likely reflects both reduced signal-to-noise ratios in the earlier time periods, and also variability in the onset times of focal attention within and across subjects relative to the button press responses; said another way, there is variability in the trial response latencies within and between subjects, as is to be expected (Paraskevopoulou et al., 2021).

**Figure 6.**
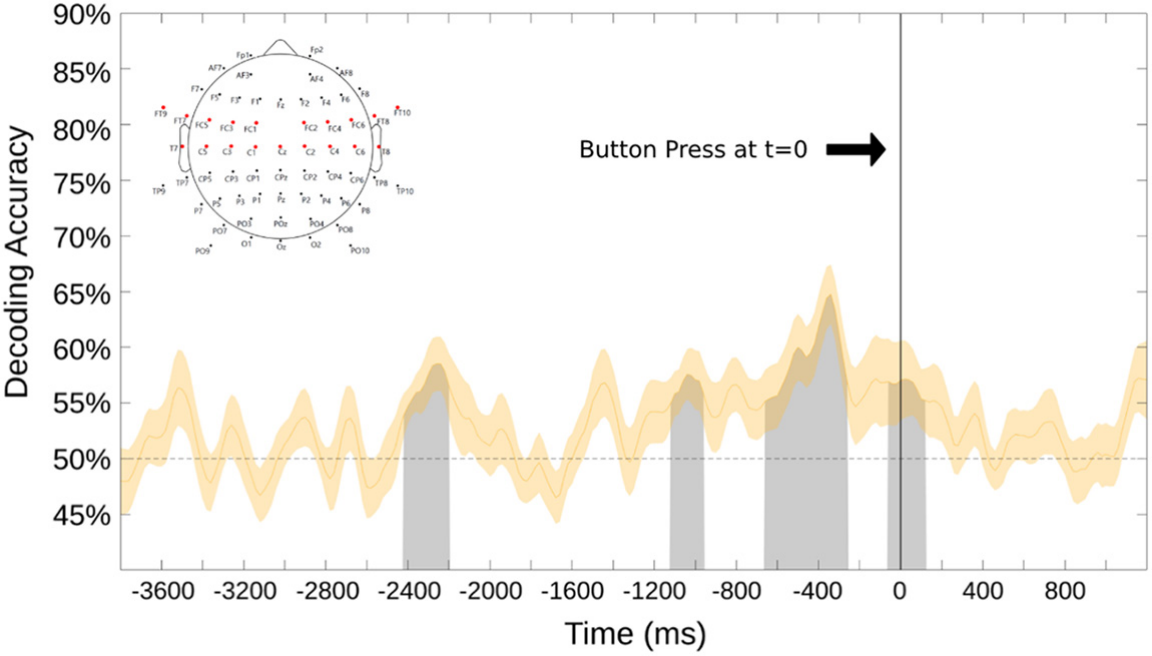
This decoding result is time locked (*t* = 0) relative to the button press signaling sustained attention to one hemifield. The shaded area surrounding the curve is the SE. This analysis was performed over 19 central and frontocentral electrodes (shown as the red electrodes in the map at top left). As in Figure 5, the black line denotes time 0, which is the recorded onset of the button press.

We performed the decoding shown in Figure 5 across occipital and parieto-occipital scalp electrodes, with the idea being to focus on posterior scalp attention-related alpha EEG signals (Worden et al., 2000; Rihs et al., 2007; Van Diepen et al., 2019). This was important to do because in our experiment, by design, motor preparation was correlated with choice attention condition; subjects pressed a leftward-pointing keyboard arrow with the right index finger when they chose to attend left, and a rightward keyboard arrow with the right middle finger when they chose to attend right. Because there is alpha frequency activity related to motor processing, referred to as the “mu rhythm,” decoding alpha, as we have done, could be tapping into motor preparation-related mu activity (Gastaut and Bert, 1954). Since the mu rhythm is generated in motor cortex and related areas, and has a central scalp distribution in the EEG (Deiber et al., 2012; Yin et al., 2016; Ross et al., 2022), we conducted a separate decoding analysis over 19 central and frontocentral scalp electrodes to investigate whether significant decoding of mu rhythm activity related to motor preparation could explain or contribute to our decoding results. Figure 6 shows decoding accuracy from the central/frontocentral scalp electrodes, using the same decoding pipeline as used for the data in Figure 5. Although there are sporadic periods of above-chance decoding over the central/frontocentral electrodes, the effect is weaker and has a different time course compared with the robust and consistent decoding of occipital alpha shown in Figure 5. It is difficult to know whether the central/frontocentral scalp decoding is reflecting motor preparation-related mu modulations or is instead simply a low signal-to-noise ratio, with volume-conducted posterior attention-related alpha signals being weakly decoded at the central sites. Regardless, however, when taken together, the results in Figures 5 and 6 suggest that mu rhythms related to motor preparation are not major contributors to our attention-related decoding of posterior alpha.

**Figure 7.**
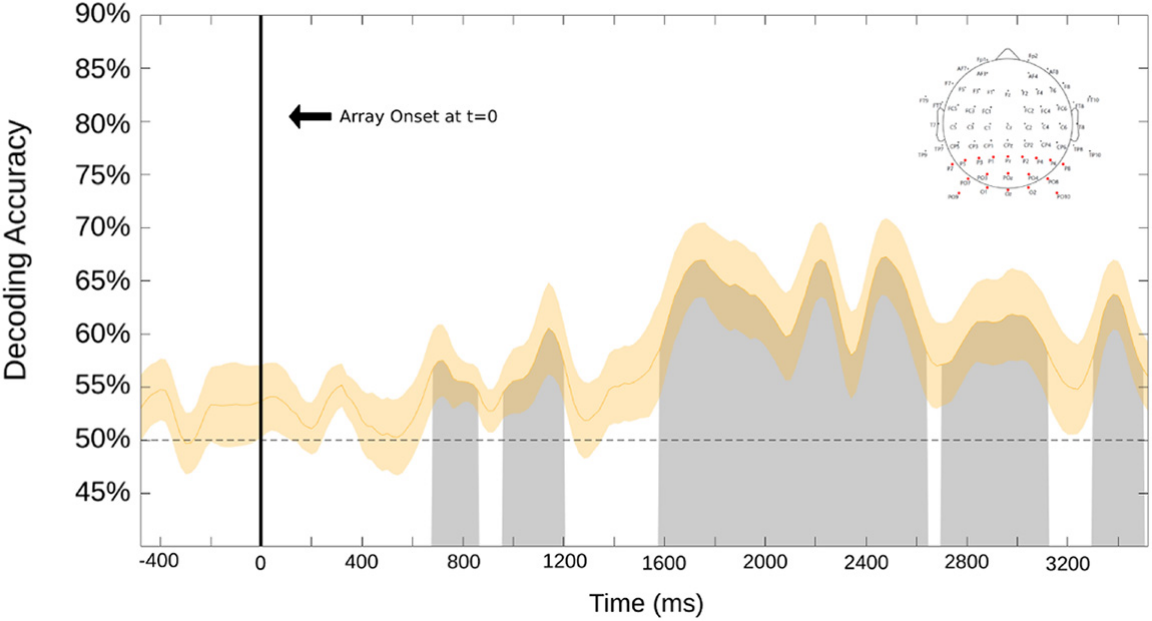
This decoding result is relative to the onset of the dot motion array sequence (represented at *t* = 0 by the vertical line). The shaded yellow area surrounding the curve is the SE. Significant time points are marked via the gray shading under the curve. This analysis was performed over 19 occipital electrodes (shown in the top right).

Another way to investigate the effects in this study is to time lock the analyses to the onset of each trial, rather than to the button press. Figure 7 shows the SVM decoding results for posterior alpha, time locked to the onset of the bilateral array. The decoding accuracy rose above chance starting at ∼800 ms after the array onset and lasted until ∼1200 ms. Following a dip in decoding accuracy, a long latency period also shows statistically significant decoding accuracy (∼1600–3500 ms after array onset). This conceptually lines up with our expectations, as we can see the buildup of decisions to attend being made, especially when noting the spread of trial response latencies (Fig. 2). The significant decoding presented here shows that a shift in volitional attention is occurring as anticipated at a time of the participant’s choosing relative to the start of the trial and can be decoded in the alpha band. In summary, these results demonstrated the capability to achieve significantly above average decoding of sustained volitional attention in a setting with minimal external influence or cuing, as well as the ability to decode the broad approximate onset of volitional spatial attention averaged across a wide range of trials without information about the onset of attentional deployments.

**Figure 8.**
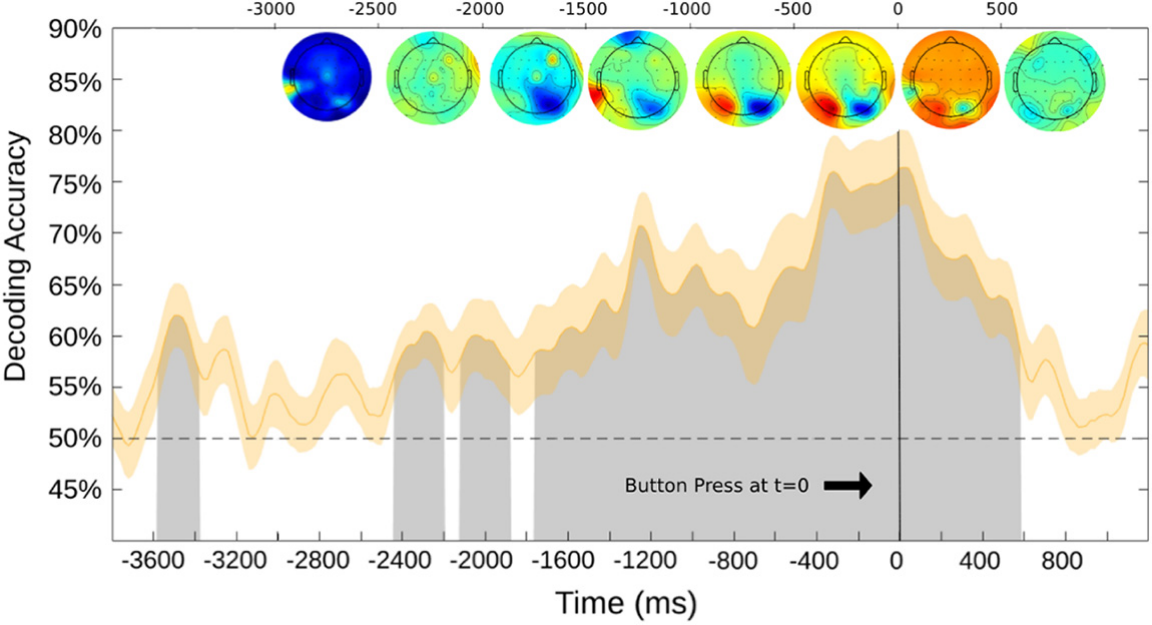
Support vector machine decoding of all electrodes (excluding mastoid channels). Corrected weight maps are above the decoding curve (in 500 ms sections). The decoding was time locked (*t* = 0) to the button press. Black line denotes time 0, which is the recorded onset of the button press.

We also conducted a bout of support vector machine decoding of all electrodes (without mastoid references), so we can compute the weight maps of which electrodes were the most impactful influences on the decoding (Fig. 8)—any deviation from zero indicates the strength of the contribution to the decoding. The weight maps show a pattern very similar to those of the topographic alpha-band voltage maps: the primary driver of the decoding stems from the posterior electrodes. This analysis adds evidence to our hypothesis that the significant decoding presented is primarily because of alpha-band oscillations in posterior electrodes as shown in prior studies of willed attention (Bengson et al., 2014).

### Current source density

**Figure 9.**
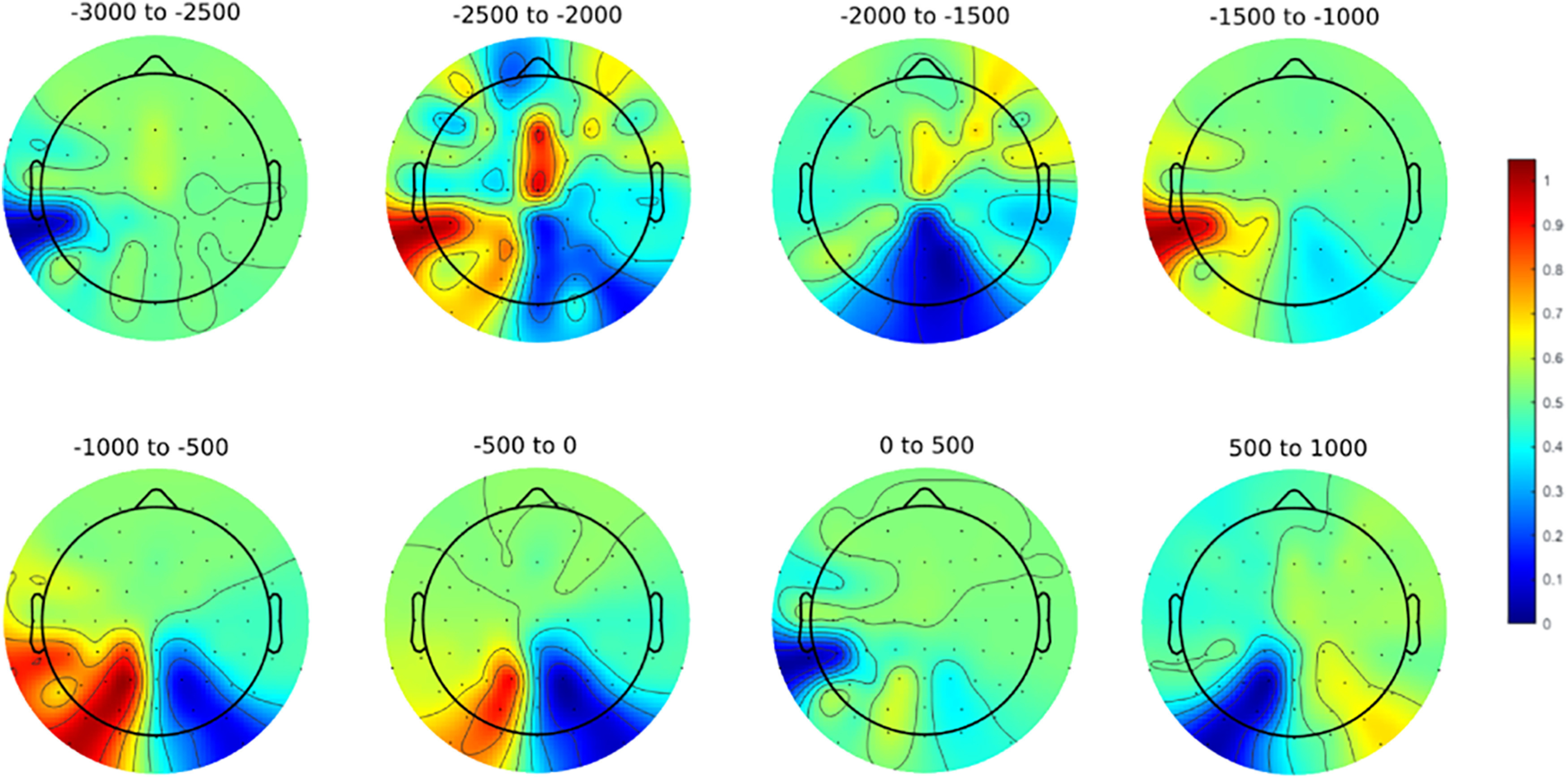
Topographic maps of the current source density within the alpha band (9–11 Hz), plotted time locked relative to the button press (in milliseconds). The color scale is based on the absolute value relative to the highest/lowest difference in value for each individual plot, thus showing where the signals primarily stemmed from for each discrete time range.

**Figure 10.**
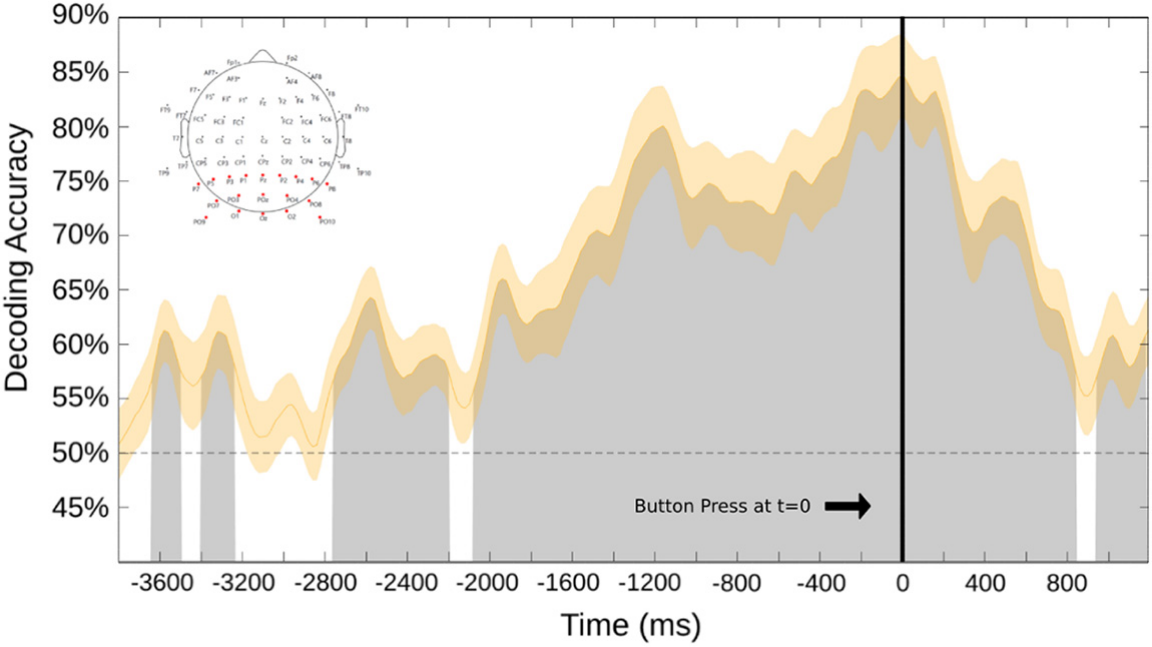
The SVM decoding accuracy at each time point, time locked (*t* = 0) relative to the button press. The data fed into the classifier is the current source density of the alpha band. The shaded area surrounding the curve in yellow is the SE. This analysis was performed over 19 occipital and parieto-occipital electrodes (shown as the red electrodes in the map at top left). Black line denotes time 0, which is the recorded onset of the button press.

**Figure 11.**
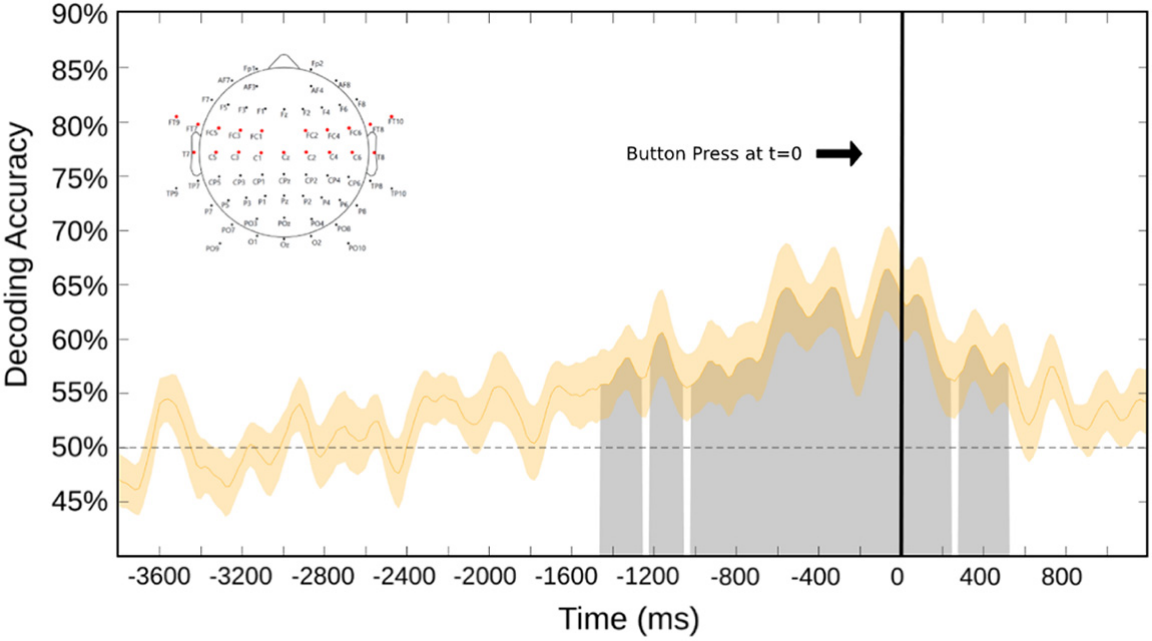
This SVM decoding result is epoched relative to the button. The data fed into the classifier is the current source density of the alpha band. The yellow shaded area surrounding the curve is the SE. This analysis was performed over 19 central and frontocentral electrodes (shown as the red electrodes in the map at top left). Black line denotes time 0, which is the recorded onset of the button press.

To further ensure that the primary signals we observe in the frequency band of interest are from the area we hypothesize, we also ran a Laplacian filter to extract the current source density (CSD) from the scalp voltages, which we then filtered to the alpha band (9–11 Hz). We have created topographic maps of the scalp CSDs, which show the alpha-band signal arising from primarily posterior electrodes at the time points of interest (Fig. 9). The current source densities also support the hypothesis that the mu rhythm is not volume conducting into the occipital channels, causing a spurious effect. To expand on this analysis, we have also conducted decoding on the posterior (Fig. 10) and central (Fig. 11) electrodes to confirm where the signals in the alpha band were stemming from.

The support vector machine analysis conducted over the occipital electrodes of the alpha-band current source density showed decoding significantly above chance for a majority of the presented time period (Fig. 10), thus showing that the occipital electrodes are the primary driver of the significant decoding presented here. In contrast, the support vector machine analysis for the central electrodes had a comparatively lower decoding accuracy, and occurred at more limited time points closer to the onset of the button press (−1450 to 500 ms). This aligns with our hypothesis that the alpha band oscillations presented in the experiment are largely derived from posterior electrodes, as is shown in former studies of willed attention (Bengson et al., 2014) and not from the mu rhythm found in central electrodes (Fig. 11).

## Discussion

In this study, we investigated voluntary visual spatial attention when guided internally by the subject’s free choices about both when and where to focus attention (i.e., self-initiated or willed attention). Prior research on willed attention asked subjects to choose where to attend, but typically in response to a prompt (Gmeindl et al., 2016) that signaled the subjects when to voluntarily focus spatial attention (Taylor et al., 2008; Hopfinger et al., 2010; Bengson et al., 2014, 2015; Liu et al., 2017; Rajan et al., 2018; Bengson et al., 2020). The act of deploying attention in the real world need not be cued externally; that is, not every shift of attention requires an explicit extrinsic temporal or spatial guiding signal (Taylor et al., 2008; Hopfinger et al., 2010; Bengson et al., 2014). In the present work, we presented subjects with bilateral, dynamic dot motion displays, asking them to view the displays, and then to spontaneously focus spatial attention on either the left or right patch at a time of their choosing, thus eliminating any explicit external attentional cue or prompt. We analyzed the EEG data using support vector machine decoding, which has been shown to be a sensitive method for quantifying the contributions of EEG alpha signals to spatial attention in prior research (Samaha et al., 2016; Bae and Luck, 2018).

We found that willed attention could be decoded from the posterior scalp EEG alpha signals. We time locked our data relative to the button press that the subjects made to signal detection of a task-relevant target; the button press also, however, signaled that covert attention has been shifted to a specific hemifield. Our analysis revealed robust, statistically significant decoding of the direction of attention from about −1900 ms before the button press, to ∼750 ms after the button press, with weaker and less consistent decoding before this period. This is the first demonstration of lateralized EEG alpha during self-generated voluntary spatial attention in the absences of cues or prompts (Fig. 4). This empirical finding has important theoretical implications for understanding voluntary attention because the shifts of attention were self-initiated, and yet were still accompanied by lateralized EEG alpha over posterior scalp, thereby providing an important test of the role of alpha mechanisms in covert visual spatial selective attention. These results provide a good foundation for future studies to directly investigate similarities and differences between cued and willed attention under conditions of willed attention that do not involve any prompts (as were used in prior work; Bengson et al., 2014).

The present study investigated not only where participants chose to attend, but also when, using uncued/prompted, dynamic visual displays. Although our methods could not provide high-temporal resolution measures of the onset of attention shifts within subjects, our results provide the very broad time course of covert shifts of attention, showing that they reliably appeared as much as ∼2 s before the overt behavioral response required in the task. As a sanity check to ensure participants were not preplanning locations to attend, we also time locked our analyses relative to the onset of each trial (instead of only to the button press), which ensured that attention was not directed before trial onset. To be clear, these analyses do not pinpoint the precise timing with which individuals shifted attention, but do provide the general time course of the shifts of attention across the population of participants relative to their behavioral responses (Fig. 5).

In the literature, a variety of influences that affect voluntary attention have been considered, including priming (Li et al., 2020), experience (Brockmole and Henderson, 2006; Goldfarb et al., 2016; Theeuwes, 2019; van Moorselaar et al., 2021), reward (Della Libera and Chelazzi, 2009; Peck et al., 2009; Hickey et al., 2010; Failing and Theeuwes, 2018; Meyer et al., 2020), object meaning (Hayes and Henderson, 2021; Peacock et al., 2021; Gayet and Peelen, 2022), and high-level behavioral goals and motivations (Serences et al., 2005; Lepsien et al., 2011; McMains and Kastner, 2011; Banerjee et al., 2015; Luck et al., 2021). Our study adds to this literature by focusing on willed attention, which, although it would be expected to interact with the foregoing, can also be considered independent of other such factors.

The study of willed attention may also be considered in relation to the extensive literature on self-generated actions have been studied in the context of motor intention (for review, see Eagleman, 2004). In this area of scholarship, a distinction has been drawn between willed and automatic control of actions, with attention being a key distinguishing component of prominent models (Shiffrin and Schneider, 1977; Norman and Shallice, 1986; Jahanshahi et al., 1995; Haggard et al., 2002; Haggard and Clark, 2003; Passingham and Lau, 2017). A core concept is that intentions arise before actions, and that the antecedent neural activity could therefore provide information about the underlying neural mechanisms of intention (Soon et al., 2013). For example, the work of Libet et al. (1982, 1983a) on motor intentions sought to reveal the neural correlates of intentions to act (Frith and Haggard, 2018). Subjects were instructed to make a volitional movement at a time of their choosing, while also reporting the time (on a clock face-like timer) of their arising intention to move. Libet et al. (1982, 1983a,b) used the reported time value as a temporal stamp that he compared with the backward averaged event-related potentials that were time locked to muscle activity preceding the movement. He found that neural activity preceded the reported time of first intention by hundreds of milliseconds. Our present study applied a similar framework as this literature on self-generated motor actions, but instead probed willed attention by backward decoding the EEG from the button press response, allowing us to decode the electrophysiological correlates of willed attention as the time period in which the decoding accuracy for left versus right choices rose above chance.

Our findings have a direct consequence for our understanding intention by moving beyond the very well studied realm of motor intentions, to the case of intentions to attend, that is, willed attention. Importantly, it demonstrates that even in cases where the subject is not prompted by a cue or prompt to make a decision about where or when to attend, fully self-generated shifts of attention have detectable neural correlates. In our EEG work, while we are unable to identify the underlying neuroanatomical correlates of willed attention, this work may motivate future research, for example, using fMRI (Bengson et al., 2015), simultaneous EEG and fMRI recording (Liu et al., 2016), magnetoencephalography (Hardy et al., 2022), or intracranial recording (Helfrich et al., 2018; Stolk et al., 2018), which would help to elucidate the underlying neural networks involved.

The approach we have used here may also be applicable in applied research, for example, in brain–computer interface (BCI) applications, where brain activity related to intentions to shift attention could be tapped to control devices by inferring intentions directly. A BCI should be built around neural signals having reliable features for feature extraction (i.e., they should reflect the subject’s intent), which would be a benefit if based on a noninvasive technique (e.g., scalp-recorded EEG), and should also have an optimal signal-to-noise ratio (Shih et al., 2012; O’Sullivan et al., 2015; Choi and Kim, 2019). Alpha oscillations elicited by a decision to attend in a willed attention setting may be such a signal, being recordable noninvasively from the scalp (as well as intracranially) and having a relatively high signal-to-noise ratio (i.e., alpha-to-ongoing EEG) for noninvasive recordings. Establishing the reliability of the alpha signal as a measure of intention without the constraint of external cuing or prompting, may provide a step forward that, together with advances in technology, hold promise for BCI applications.

## Synthesis

Reviewing Editor: Anne Keitel, University of Dundee Decisions are customarily a result of the Reviewing Editor and the peer reviewers coming together and discussing their recommendations until a consensus is reached. When revisions are invited, a fact-based synthesis statement explaining their decision and outlining what is needed to prepare a revision will be listed below. The following reviewer(s) agreed to reveal their identity: Christopher Gundlach. Note: If this manuscript was transferred from JNeurosci and a decision was made to accept the manuscript without peer review, a brief statement to this effect will instead be what is listed below.

The reviewers and editor agreed that the manuscript has improved and thank the authors for their thorough revision. However, Reviewer #2 pointed out, and the editor agrees, that the strong conclusions in the manuscript are not justified. This concerns two main points:

1) The study cannot make claims about comparisons between conventional cued paradigms and willed-attention designs.

2) The paradigm does not allow to pinpoint the moment of an attention shift.

Reviewer #2 added during our discussions: “the study can only be seen as a starting point leaving open all the imminent questions: are self-paced willed and cued, willed attention comparable? What are the temporal dynamics of willed attention? etc. I in general see that the researchers are tackling a relevant research question. Rephrasing the current findings would allow stressing the point that there are still some unanswered questions regarding cued and willed attention and that new paradigms (such as the one presented) may be of use for tackling these questions.”

These concerns mean that the current study should be framed as a “step in the right direction”, with a more humble wording and toning down of the implications of the study.

Please see below for detailed, unabridged comments made by the reviewers. Please respond to the comments in a point-by-point manner.

*****

Reviewer #1 - Advances the Field (Required)

The manuscript addresses a significant gap in the previous literature related to the alpha power in anticipation of spontaneous orienting of attention, in contrast to endogenous or exogenous orienting.

Reviewer #1 - Comments to the Authors (Required)

The manuscript in its present form is clear and interesting. I compliment the authors for their job. All my previous concerns have been addressed. I think the manuscript improved significantly.

Reviewer #2 - Advances the Field (Required)

I have problems grasping the conceptual relevance of the manuscript, which became even more evident with the re-submission: what is the actual question and aim of the study? It is neither designed to test differences between willed and conventionally cued spatial attention nor does it allow to pinpoint the temporal dynamics of willed attention, i.e. there is no unambiguous interpretation of the early, attention-onset related signals. The data shows that alpha-band activity is lateralized during sustained top-down attentional selection of stimuli relevant to a behavioral task.

Reviewer #2 - Comments to the Authors (Required)

The Temporal Dynamics of Willed Attention in Vision

The authors with a lot of effort addressed points raised in the previous reviews. This is much appreciated. Some points were addressed quite convincingly: for instance the additional analyses aimed at disentangling motor preparation related from willed attention-related processes rather strongly suggest that motor-related processes may not strongly contribute to the signals picked up by the decoding. There are, however, a few very critical points.

It is still not totally clear to me to fully grasp the conceptual level of the study. While the authors correctly and convincingly discuss that attention-related neural processes are often confounded with cue-processing processes in conventional attentional cueing designs, the current study seems to fall short of the strong claims the authors make.

For instance, the study was not planned to directly compare conventional cueing designs and the current willed-attention design, as there is no conventional cueing condition implemented in the current experiment. This would in principle allow for a direct comparison of cognitive processes and related neurophysiological measures such as alpha-band activity.

The authors also still claim that they pinpoint the time “when the subjects allocated their spatial attention.” (lines 86 and 87 or lines 12 onwards and 28 onwards, line 429 onwards. line 458 onwards). Such a claim, is, however, hard to substantiate with the current design, as there is no ‘objective’ measure to pinpoint the start of attentional allocation. There are rather some constraints of the task per se, as the authors clarified: participants should attend to one stimulus and track the changes of at least one cycle. This would mean that in each trial participants should have attended a respective stimulus for at least 2000 ms before each button press, given the stimulus display. As visible in Figures 5 and 8 decoding this attentional state is possible for almost all of the 2000 ms + mean reaction time of 200 ms before each button press. This is an effect that has been shown before (e.g. Samaha et al., 2016). Before that period the exact onset of willed attention should vary across trials. With this in mind I still can’t figure out what a meaningful interpretation of the time-resolved decoding analysis averaged across trials would be. The decodability for time-points before around 2200 ms should depend a.) on the number of trials for which participants already attended to the stimulus they later responded to (i.e. for which the SVM grouping variables attend left vs right are indeed valid) and b.) on the decodability of the two grouping variables per se. The further you go back in time the lower the decoding performance should be, as there are fewer and fewer trials in which participants already paid attention and the label of the grouping variable used for decoding is correct. With this in mind, I don’t see what can be extrapolated from here, as the design neither allows to pinpoint the onset in each trial nor does it allow to somehow quantify whether the ‘strength’ of the attentional selection varies across the time course of each trial. I still don’t see the point in the time-resolved analysis. Related to the goals of the study, to identify where and when subjects allocated spatial attention, as the authors stated in line 85 onwards, the study can’t say anything about the when of spatial attention that stretches beyond the constraints of the specific task (“attend to the stimulus for at least one cycle/2000 ms”). The data shows that alpha-band activity is lateralized during sustained top-down attentional selection of stimuli relevant to a behavioral task. A finding that was extensively shown in the literature.

I also don’t see how the “willed attention paradigm eliminates all of these potentially confounding issues...” (line 75 onwards) by sacrificing an estimation of the exact onset of attentional shifting.

Minors:

- Line 166 onwards: how many trials entered the analysis per subject?

- The histograms of Figures 2 and 3 could be more fine-grained to reveal more detail in the relevant portion of the data

- Given the cycle of 2000 ms for stimuli on both sides, how can reaction times of more than 1 s be unambiguously allocated to one stimulus (the histogram, figure 3, reveals even responses at almost 2s)?

- Figure 8: What values and units do the colors of the topographies represent?

- Figure 9: What’s the unit of the color bar?

- Line 386 onwards: “far less significant” hints at the interpretation of the p-value concerning an effect size; the level of significance does however not say anything about the size of an effect. Maybe better “decoding accuracy was smaller.” Also: differences were not formally statistically tested.

- Line 420 onwards: What’s the new view on electrophysiological correlates of attention?
